# Mutagen-induced somatic mutation rate in primary mammalian cells in relation to maximum life span

**DOI:** 10.70401/geromedicine.2026.0023

**Published:** 2026-05-08

**Authors:** Johanna Heid, Shixiang Sun, Julia Ablaeva, Moonsook Lee, Zhengdong Zhang, Vera Gorbunova, Andrei Seluanov, Alexander Y. Maslov, Jan Vijg

**Affiliations:** 1 Department of Genetics, Albert Einstein College of Medicine, Bronx, NY 10461, USA.; 2 Department of Biology, University of Rochester, Rochester, NY 14627, USA.; 3 Department of Medicine, University of Rochester Medical Center, Rochester, NY 14627, USA.

**Keywords:** Somatic mutations, species-specific lifespan, mutation burden, N-ethyl-N-nitrosourea, DNA repair

## Abstract

**Aims:**

Testing the hypothesis that excess mutations induced in primary fibroblasts by a low dose of N-ethyl-N-nitrosourea (ENU) are inversely correlated with species-specific maximum life span.

**Methods:**

To measure excess mutations induced by ENU we treated primary cells of 10 mammalian species, greatly differing in life span. We treated all cells with a low dose, non-toxic dose of ENU (20 ug/ml). We then extracted DNA from all treated and untreated cells and quantified somatic mutation burden by single-molecule sequencing. We measured excessive mutations by calculating the ΔSNVs and we analyzed this across species with linear regression.

**Results:**

The average values for ΔSNV were found to range from 0.773 in mice to 0.367 in whale, resulting in a modest inverse correlation with species-specific maximum life span (*R*^2^ = 0.2067, *P* < 0.001).

**Conclusion:**

We conclude that DNA repair accuracy, the main determinant of genome sequence integrity, modestly correlates with life span suggesting that longer lived species have better repair capacities compared to shorter-lived species, which is in keeping with genome instability being a primary hallmark of aging and highlights its important role for longevity.

## 1. Introduction

Genome instability, i.e., the tendency of the genome to undergo alterations, is considered a primary hallmark of aging^[1]^. This goes back to Failla^[2]^ and Szilard^[3]^, who independently proposed, shortly after the elucidation of the DNA structure by Watson and Crick^[4]^, that aging could be logically explained by the accumulation of mutations in somatic cell genomes. At that time, neither the source of mutations nor methods to analyze somatic mutations were known. Once the mechanism of DNA replication was elucidated^[5]^, and especially after the discovery of DNA repair^[6]^, mutations could be attributed to errors during replication or repair of damaged DNA templates. It is now known that spontaneous DNA damage occurs at a very high frequency, with the numbers of DNA lesions, such as breaks and loss of bases, possibly as high as 100,000 per cell per day^[7]^. Almost all this damage is quickly removed, but not without errors. The errors are the cause of mutations, alterations in the genome sequence information. Initially, the burden of such mutations is low, but using new methods for detecting somatic mutations, it has now been shown that mutations can accumulate to high levels in mouse and human tissues during aging^[8,9]^.

Mutations are essential for generating the genetic variation as the substrate of evolution, and mutation rates cannot be reduced to zero without incurring a high fitness cost^[10,11]^. Germline mutation rates have long been calculated from alterations at specific loci between parents and offspring, for example, in humans from the frequency of affected children of unaffected parents^[12]^. In contrast, *de novo* mutations in somatic tissues are distributed across individual cells or small clones and are therefore highly diluted in bulk DNA samples. As a result, they cannot be reliably detected by conventional sequencing and require single-cell or single-molecule approaches^[8,13]^. Alternatively, sequencing of clonal populations can be used as a surrogate for single cells analysis. Cagan *et al*. used this approach to analyze age-related accumulation of somatic mutations in intestinal crypts from 56 individuals representing 16 different mammalian species and found an inverse correlation between somatic mutation rate and species lifespan^[14]^.

While these results greatly strengthen the idea that DNA repair is a determinant of life span, it does not directly measure the accuracy of DNA repair. DNA repair accuracy should be distinguished from DNA repair capacity to quickly remove DNA damage. Indeed, quick repair would promote survival but possibly at the cost of errors, which become manifest as the somatic mutations that cause cancer and, possibly, age-related functional loss and diseases other than cancer^[10]^.

Reasoning that somatic mutation rates are determined by the accuracy of DNA repair, we previously used single-cell whole genome sequencing to measure spontaneous and bleomycin-induced somatic mutations in primary fibroblasts of several rodent species with markedly different lifespans, including mouse, guinea pig, blind mole rat, and naked mole rat. In that study, mutation burden following bleomycin treatment showed an inverse correlation with species life span^[15]^. Here, we greatly expand this work using our recently developed single-molecule mutation sequencing assay (SMM-seq)^[16]^, which enables accurate detection of low-frequency somatic mutations at scale. We present data on ten mammalian species using the powerful point mutagen N-ethyl-N-nitrosourea (ENU) as a challenging agent. ENU is a simpler agent than bleomycin and induces only base substitutions^[17]^. It has also been very well characterized^[18]^, and as a direct acting agent, is not susceptible to possible species differences in metabolization. The results support an inverse correlation between ENU-induced mutation burden and species lifespan, with several caveats, which are discussed. Our approach provides insight into species-specific differences in genome maintenance and mutational processes.

## 2. Methods

### 2.1 Animal species

Compliance with ethical regulations and all animal experiments were approved by the University Committee on Animal Resources of the University of Rochester. Cells from young adult, wild animals were sourced as previously described^[19,20]^, with the exact age of the animals mostly unknown. Mice were purchased from Jackson Laboratories. Hamsters and rats were purchased from Charles River Laboratories. Chinchillas were purchased from Moulton Chinchilla Ranch. Naked mole rats were from the colony at the University of Rochester. Bowhead whale data was derived from our earlier publication^[19]^.

### 2.2 Cell isolation and culture

Primary fibroblasts were obtained from lung or skin of each species. Commercially available human lung fibroblasts (IMR90) were obtained via ATCC (CCL-186). Human cells were cultured in DMEM supplemented with 10% FBS and 1% PenStrep (all Gibco). Cells were maintained in 10% CO_2_ and 3% O_2_ at 37 °C. Primary skin and lung fibroblasts from the different mammalian species were isolated and cultured as described previously^[19,20]^ and early passage cells from cryo-preserved vials were used for the experiments. In brief, cells were cultured in EMEM supplemented with 20% FBS and 1% PenStrep (Gibco) at 37 °C at 5% CO_2_ and 3% O_2_, except for cells from NMR, which were maintained at 32 °C.

### 2.3 Mutagen treatment

A stock solution of ENU (Sigma-Aldrich) 100 mg/ml in 100% ethyl alcohol was used to prepare medium immediately prior to application. If not otherwise stated, confluent cells were treated overnight with 20 μg/ml ENU, washed twice with PBS, and a new medium was added. Confluent cells were then split 1:4 into a 10-cm dish and grown in regular medium until confluence. Using this approach, cells will have doubled approximately twice when collected regardless of their proliferation rate. Viability was assessed using the Guava ViaCount assay (Cytek 4000–0040) and apoptosis rates were determined with the Guava Nexin Assay (Cytek 4000–0450). Both assays were run on a Guava EasyCyte flow cytometer.

### 2.4 Library preparation and sequencing

Genomic DNA (gDNA) was isolated from frozen cell pellets using the Quick DNA/RNA Microprep Plus Kit (Zymo D7005) according to the manufacturer’s instructions and quantified with a Qubit kit (Thermo Fisher Scientific). We used 300 ng of DNA for library preparation as described previously^[16]^. Briefly, gDNA was digested with the restriction endonuclease AluI (New England Biolabs (NEB)), and the resulting fragments were end-repaired and ligated to custom hairpin-shaped adapters using the NEBNext Ultra II DNA Library Prep Kit for Illumina (NEB). The ligated material was then subjected to size selection on a PippinHT instrument to isolate the 500–800 bp fraction, and the selected material was used for rolling circle amplification with SD polymerase (Bioron). Indexing was performed using the NEBNext Ultra II Q5 master mix together with the NEBNext Multiplex Oligos for Illumina (NEB) to generate the final SMM-seq libraries. Library quality was assessed with a TapeStation (Agilent) and quantified with Qubit (Thermo Fisher Scientific). Sequencing was performed by Novogene (USA) on NovaSeq 6000 and NovaSeq X Plus platforms in 150 bp paired-end mode.

### 2.5 Computational analysis and variant calling

Raw sequencing reads were adapter and quality trimmed before alignment to the corresponding reference genome. Subsequent sequencing analysises were performed as described^[16]^, using the following tools: Python v.2.7.18, TrimGalore v.0.4.1, BWA v.0.7.13, Samtools v.1.9, Picard v.1.119, GenomeAnalysisTK v.3.5, Bcftools v.1.9, and tabix v.0.2.6. Mutations were called using SMM (https://github.com/msd-ru/SMM). Germline variants were distinguished from somatic mutations by additional filtering steps after alignment to the reference genome^[16]^. To humanize the mutation counts from the animals, we calculated the ratios of trinucleotide frequencies between animals and humans and applied these ratios to normalize the mutation counts. Analysis of mutational spectrum and signatures were conducted in R v.4.3.3, with MutationalPatterns v.3.12.0. A known ENU signature was obtained from Kucab *et al*.^[21]^, and known mutational signatures were obtained from the COSMIC database (https://cancer.sanger.ac.uk/signatures/).

### 2.6 Statistical analysis

Graphs were generated and statistical analyses were performed using GraphPad Prism and R. Comparisons between two independent groups were conducted using unpaired two-tailed Student’s *t*-tests (or Welch’s *t*-tests when variances were unequal). For paired comparisons between control and ENU-treated samples, Wilcoxon matched-pairs signed-rank tests were applied. One-way or two-way analysis of variance was used for analyses involving more than two groups, followed by Tukey’s or Šídák’s multiple comparisons tests where appropriate. Simple linear regression was used to assess the relationship between mutational burden and Maximum Life Span (MLS), body weight, or longevity quotient (LQ). To assess the robustness of the relationship between mutational burden and MLS, leave-one-out sensitivity analyses were performed by iteratively excluding human, whale, or rat and re-evaluating the correlation.

## 3. Results

### 3.1 ENU treatment conditions

**Figure 1.**
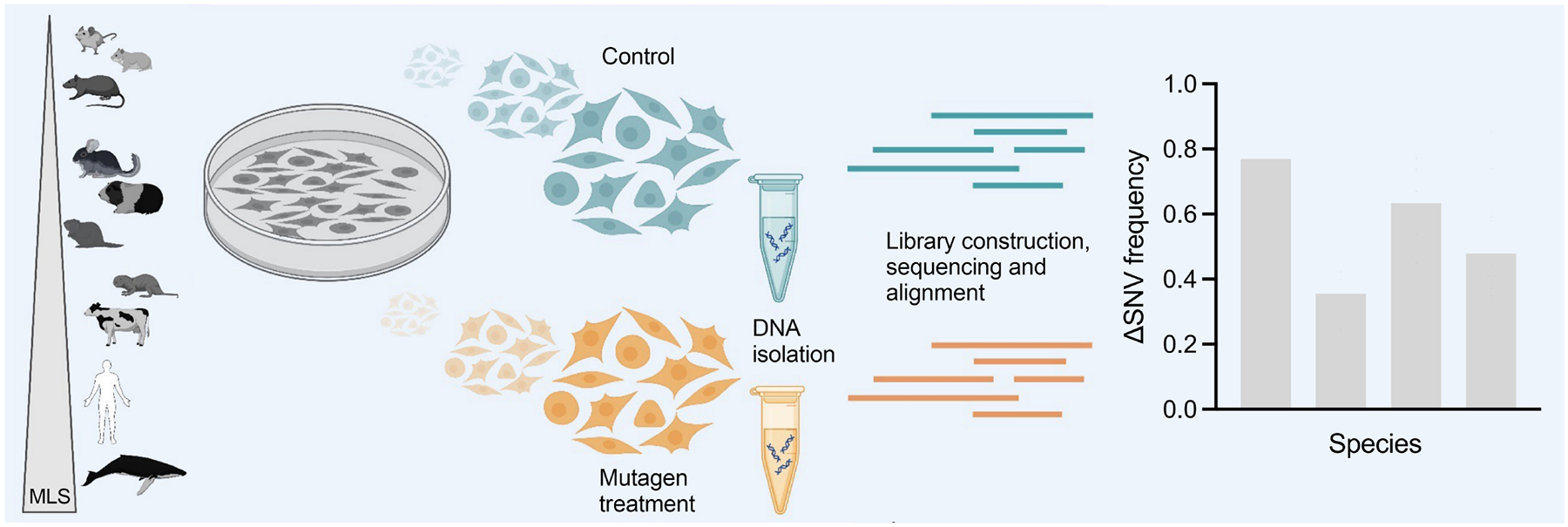
Schematic depiction of the experimental procedure of comparing primary fibroblasts of different species for excess mutations induced by the mutagen ENU. DNA extraction of control (green) and treated (orange) samples is followed by library preparation and subsequent sequencing to analyze mutational load and calculate ΔSNV as a measure of the DNA repair accuracy. ΔSNVs are then compared between species in the hypothetical example on the right. ENU: N-ethyl-N-nitrosourea; SNV: single nucleotide variant.

Somatic cells of aging organisms accumulate mutations due to constant exposure to endogenous and exogenous mutagens. To test if cells of long-lived animals accumulate less mutations upon mutagenic treatment than cells from short-lived animals, we previously used the mutagen bleomycin and did indeed find an inverse correlation of bleomycin-induced mutation rate and species life span^[15]^. However, bleomycin induces a variety of mutation types, including base substitutions, small deletions, and larger genome structural variants^[22]^, and only a small number of rodent species were tested. Here we used the mutagen ENU, a powerful point mutagen that induces mostly base substitutions^[17]^. ENU offers the advantage that it is a direct acting agent, which does not require metabolization, and induces only base substitutions. The approach is graphically depicted in Figure 1.

First, we determined optimal treatment doses and conditions using human IMR90 primary fibroblasts. Our main consideration was to attain a maximal ENU-induced mutation burden at high cellular survival. In this way, we would come close to testing a cell population’s capacity to prevent mutations through a high level of DNA repair accuracy. Of note, while there is evidence that DNA repair capacity correlates with species life span^[19,20,23,24]^, repair accuracy has never been tested on cells from so many species.

**Figure 2.**
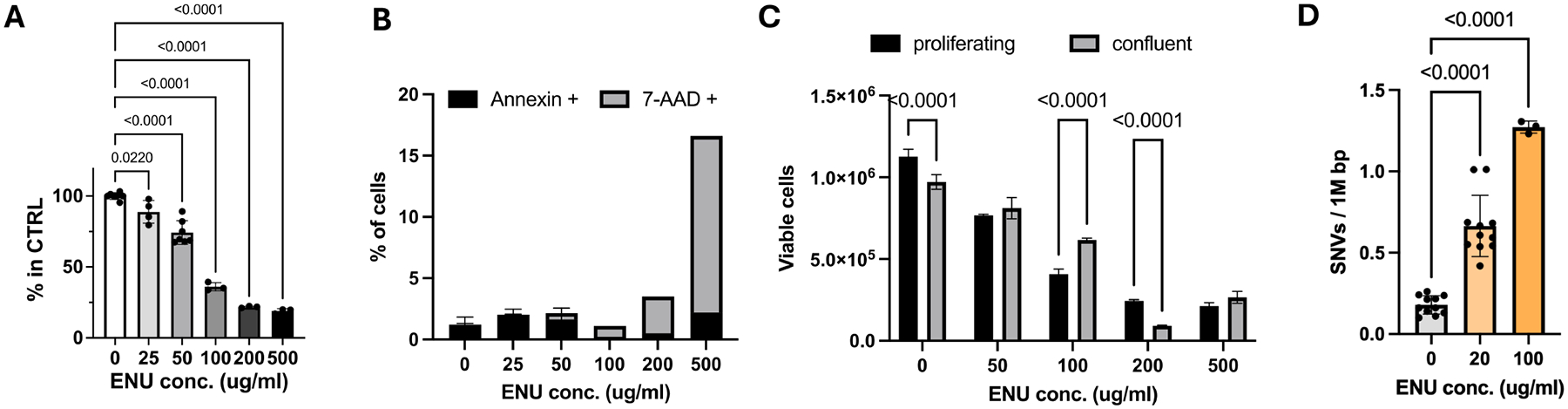
(A) Viability of human IMR90 cells after treatment with different doses of ENU. Proliferating cells were treated with ENU doses ranging from 25–500 μg/ml and the number of viable cells was assessed 72 hrs after treatment as a percentage of the control population (1-way ANOVA, *R*^2^ = 0.9747); (B) Percentage of cells in early (Annexin positive) and late stage (7-AAD) apoptosis; (C) Viability of ENU-treated cells when confluent; (D) SNV frequency in human IMR90 cells after treatment with 20 or 100 μg/ml (1-way ANOVA, *R*^2^ = 0.9259). IMR90: Institute for Medical Research-90; ENU: N-ethyl-N-nitrosourea; ANOVA: analysis of variance; 7-AAD: 7-aminoactinomycin D; SNV: single nucleotide variant.

To test the effect of ENU on cell survival, we performed a dose response analysis with proliferating human IMR90 lung fibroblasts (Methods). The numbers of viable cells were determined at 72 hrs after ENU treatment. The results showed that, as expected, viability declined with increasing dose of ENU (Figure 2A). This decline in cell viability is likely due to a dose-dependent increase in apoptotic rate (Figure 2B). Even as low as 25 μg/ml, a decline in viable cells and a slightly elevated level of apoptotic cells were still observed, but both were marginal. From these results, we concluded that depending on the mutation burden induced at this dose, results would not be confounded much by toxicity.

Nevertheless, we tested whether treating cells at confluency would reduce toxicity even further. We tested this with doses varying from 50 to 500 μg/ml ENU. The results indicated that viability was indeed higher when treating cells at confluency (Figure 2C). Based on these results, we opted for the lowest dose, i.e., 20 μg/ml ENU. Figure 2D shows that at this dose we can still detect increased mutational burden utilizing the SMM-seq method^[16]^. In the final protocol, cells were treated at confluency with 20 μg/ml ENU, then split and grown further to confluency. The latter would ensure that all cell populations had completed one or more population doublings, which would allow errors in replication-dependent DNA repair to be detected as a mechanism for mutation generation.

### 3.2 ENU-induced mutation burden in primary fibroblasts as a function of species-specific life span

**Figure 3.**
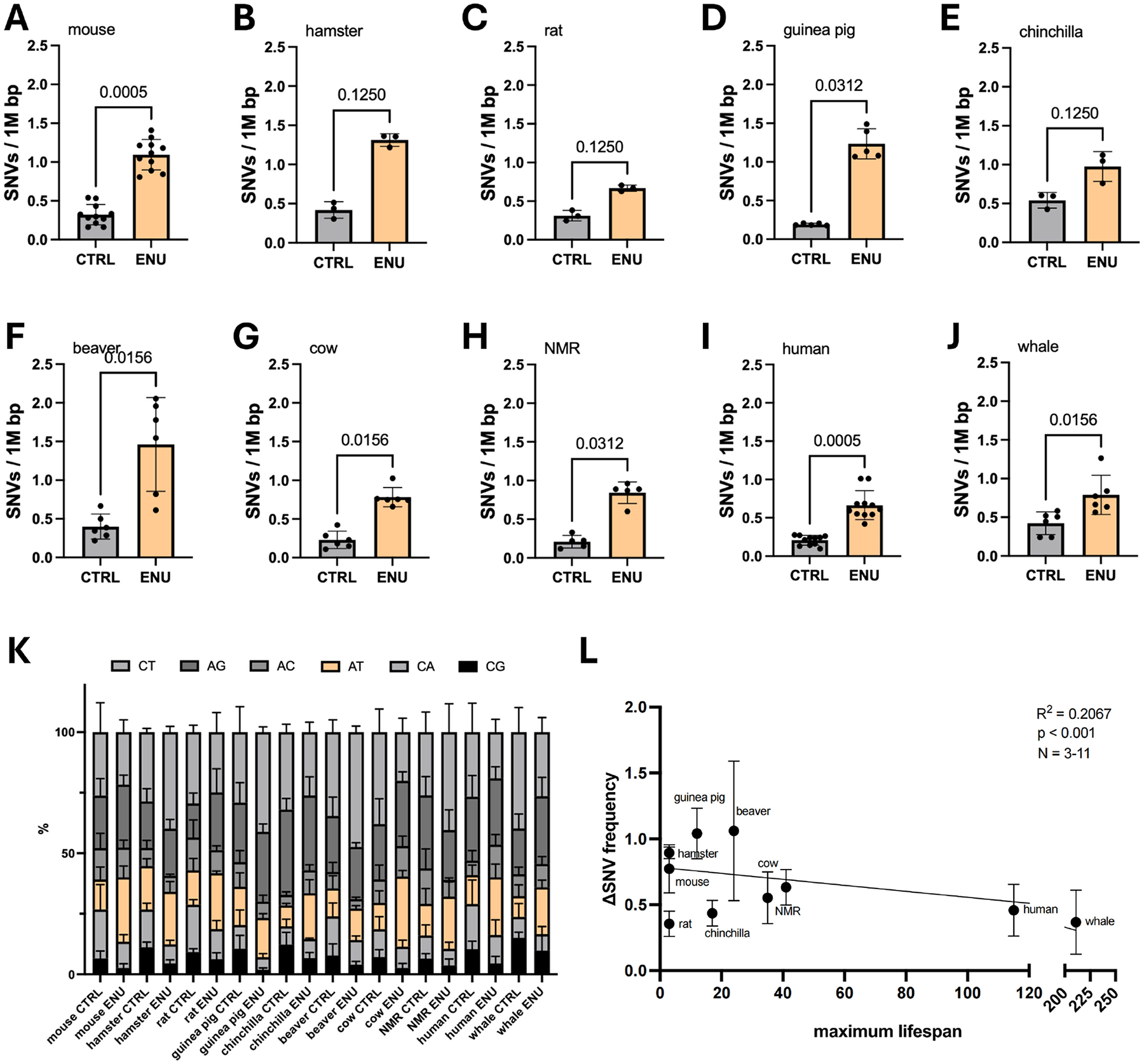
(A-J) ENU treatment at the low dose of 20 μg/ml increases the mutational load across 10 species, i.e., mouse (A), hamster (B), rat (C), guinea pig (D), chinchilla (E), beaver (F), cow (G), NMR (H), human (I) and whale (J); (K) The A > T mutations (orange) are increased across species; (L) Correlation of ΔSNV with lifespan results in a moderate negative correlation. ENU: N-ethyl-N-nitrosourea; NMR: naked mole rat; SNV: single nucleotide variant.

Once we determined optimal treatment conditions for the primary cells, we tested ENU-induced mutation burden in primary fibroblasts from animals with maximum lifespans ranging from 3 years (mouse, hamster, and rat) to 120 years (human). We also added the data from our earlier publication for the bowhead whale, which has a maximum lifespan of over 200 years^[19]^. In all cells treated with ENU, we observed an increase in mutations (Figure 3A,B,C,D,E,F,G,H,I,J). Furthermore, we observed an increase in A > T transversions in all ENU-treated samples (Figure 3K).

We next calculated the amount of single nucleotide variants (SNVs) induced by ENU, i.e., excess mutations (ΔSNV frequency) for each species and correlated that with maximum lifespan reported in the literature (Table S1). The results indicate a modest negative correlation of SNV frequency with maximum lifespan (*R*^2^ = 0.2067; Figure 3L). This negative correlation remained consistent in leaving one out sensitivity analyses after excluding human, whale, or rat (Figure S1A,B,C). We also correlated ΔSNV with body weight but found a weaker negative correlation (*R*^2^ = 0.0908; Figure S1D). Correlating ΔSNV with LQ for each species did not show any correlation (*R*^2^ = 0.03613; Figure S1E).

### 3.3 Mutational signatures

**Figure 4.**
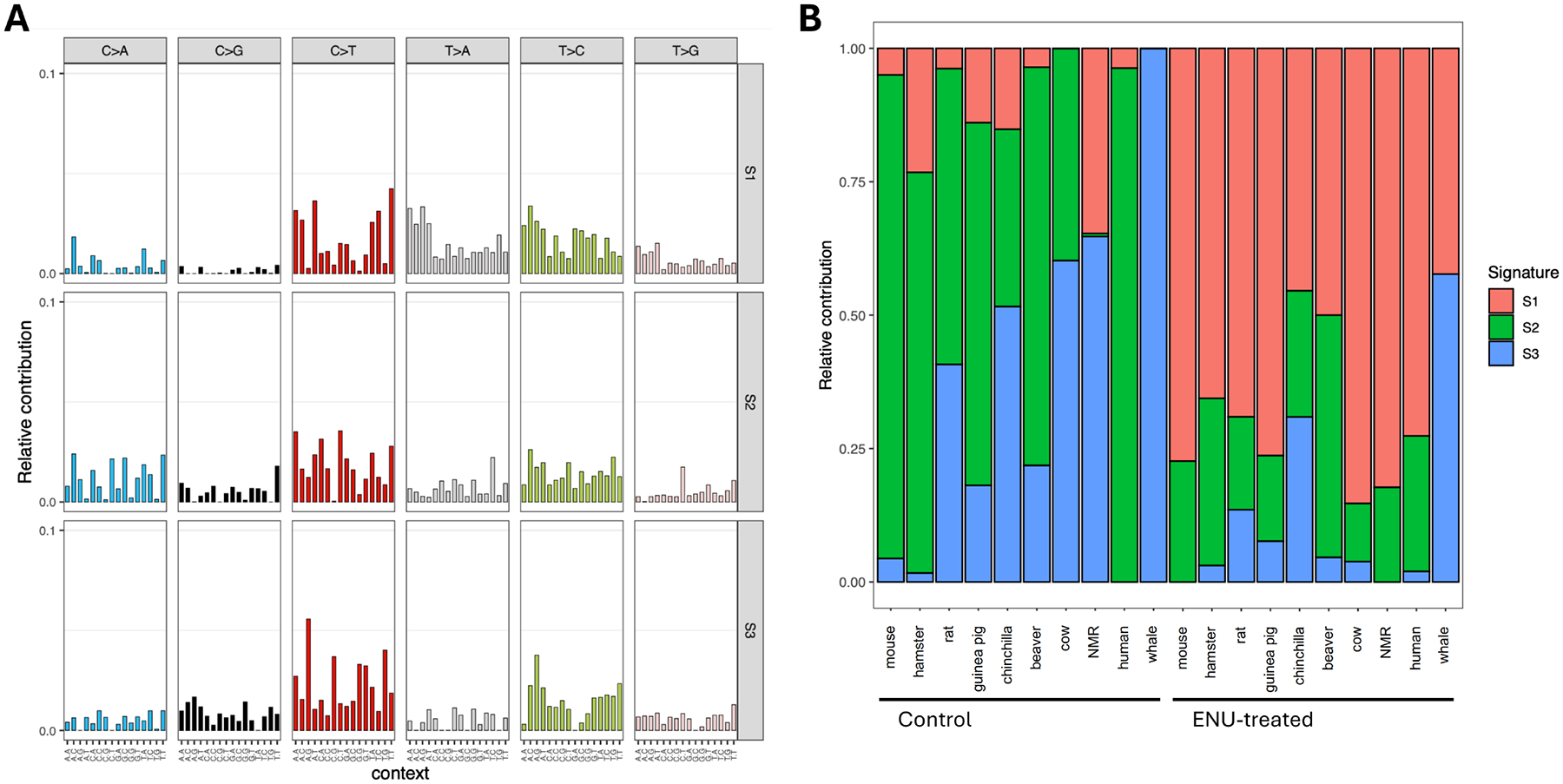
(A) Identification of mutational signatures across species resulted in three *de novo* signatures, termed S1, S2, and S3; (B) The contribution of the three signatures for all surveyed species between control and ENU-treated groups. ENU: N-ethyl-N-nitrosourea.

To analyze the impact of ENU, we combined all somatic mutations detected within each species and grouped them into control and treatment groups to visualize the mutational spectra (Figure S2). As trinucleotide composition differs substantially across species and ENU-induced mutagenesis is strongly sequence-context dependent, raw mutation counts or ENU signatures are not directly comparable across species without accounting for background trinucleotide frequencies. Therefore, for cross-species analysis, we humanized the somatic mutation counts by normalizing them to the trinucleotide frequencies in the human reference genome (Methods). We then extracted three *de novo* SNV mutational signatures, using non-negative matrix factorization, from the mutation spectra of the ten species, analyzed separately in control and treatment conditions (20 groups in total). We then compared these three signatures (Figure 4A) to those in the COSMIC database and the known ENU signature^[21]^ and found that our S1 signature is closest to the ENU signature (cosine similarity = 0.833). We observed a significantly higher contribution of S1 in the ENU treatment group (66.6% ± 15.5%, *P* < 0.001, paired Student’s *t*-test; Figure 4B). S2 resembles an aging-associated signature, including SBS5 (cosine similarity = 0.878) and SBS40 (cosine similarity = 0.887). S3 is likely to be related to SBS1 (cosine similarity = 0.609; clock-like signature) or SBS6 (cosine similarity = 0.656; associated with defective DNA mismatch repair) because of a high proportion of CpG > T mutations (Figure 4A). The proportion of S3 showed a positive association with species lifespan. However, the human signature turned out to be an outlier showing only signature S2. After excluding the human species, the life span-related S3 signature reached statistical significance (*P* = 0.003, Spearman’s rank correlation). This association remained robust when mutational signatures were re-extracted using only the control groups (signature N1 in Figure S3A,B). It is unclear why the human signature stands out and is most similar to the mouse signature.

## 4. Discussion

Age-related somatic mutation accumulation rate has been found inversely correlated with species-specific MLS^[14]^, confirming a role for DNA damage as a universal cause of aging^[25]^. Reasoning that mutations arise as a consequence of errors in DNA repair and replication, we previously tested if mutagen-induced somatic mutations were also inversely correlated with life span. Using primary fibroblasts from a limited number of rodent species, we previously found that mutation burden after treatment with bleomycin was indeed generally higher in shorter-lived species^[15]^. Here we used fibroblasts from ten mammalian species with a broad range of maximum lifespans. Cells were treated with the powerful point mutagen ENU instead of bleomycin, because as a simple, direct acting agent, ENU only induces base substitutions, which limit confounding factors. As in the previous study, a modest negative correlation between ENU-induced mutation burden (ΔSNV) and species-specific life span was observed (Figure 3L, raw and ΔSNV are provided in Figure S4A,B). As expected, an ENU signature characterized by A to T transversions could be observed in all treated samples (Figure S4C). These results indicate that cells from long-lived species have more accurate DNA repair than cells from shorter-lived species. This is in keeping with evidence that DNA repair capacity after mutagen exposure is higher in longer-lived species^[23]^. Thus far, DNA repair accuracy of somatic cells has never been analyzed. Our results are also in keeping with genome instability as a primary hallmark of aging^[1]^. However, there are several caveats in our study that should be mentioned.

First, the observed negative correlation was only modest due to some individual differences between animals but also outliers, most notably the low ENU-induced mutation burden in cells from the rat. Rats live about as long as mice, and we expected to see an ENU-induced mutation burden about equally high as observed in mouse or hamster cells. It occurred to us that the differences in body weight could possibly explain this since rats are about 10 times heavier than mice and hamsters. This would correspond to a much larger number of somatic cells in rats, which may, therefore, need better DNA repair to prevent too many mutations associated with the much higher number of cell divisions. However, when plotting the ΔSNV frequencies against bodyweight, a negative correlation with body weight was poor and ENU-induced mutations in rat cells were still more in the range of human cells.

We cannot rule out the possibility that individual differences in ΔSNV frequencies within a species are due to age differences. Indeed, somatic mutations increase with age in mice and humans^[8]^ and are likely to do so in other species as well. Unfortunately, we do not have accurate age information on animals caught in the wild. While these were all young adult animals as far as we can tell (aging in the wild occurs rarely), an exact determination of their age was not possible.

Second, our study was limited to only one mutagen and single-nucleotide mutations. While this makes the results easier to interpret, endogenous mutagens, such as reactive oxygen, can induce a range of mutation types, including genome structural variants that can currently not be accurately analyzed. Methods to do this are under development, and future expansion of this study should include different mutagens, inducing not only SNVs but also small insertions and deletions as well as larger genome structural variants. A broader range of mutagens would cover more DNA repair pathways to generate mutations and, therefore, increase the sensitivity of the assay. For example, it is possible that rat cells are less efficient than cells from longer-lived species in preventing small deletions or larger genome structural variation^[26]^. For such future studies, it will be necessary to also study the levels of DNA damage, e.g., using Whole Genome Amplification (WGA)^[27]^. While ENU is a simple, direct acting agent (which is why we selected it for this study), most other agents are more complicated and may be subject to metabolization or differences in cellular uptake. To address this question, one could think of employing a method that measures DNA damage directly, such as the well-established comet assay^[26,28]^ or a recently published method that uses a modified protocol for Single Cell Whole Genome Amplification (LCS-WGA)^[27]^.

Finally, it should be mentioned that differences in the quality and completeness of species-specific reference genomes could have influenced our variant calling results. In some cases, lower-quality assemblies contain a high proportion of unresolved regions, leading to reduced mapping rates or extensive regions with poor alignment quality. This, in turn, may result in fewer confidently called variants, particularly if the surveyed regions are biased toward gene regions or regulatory elements, which are typically under stronger DNA repair surveillance and thus accumulate fewer mutations. These factors introduce a potential bias in mutation burden and spectrum comparisons across species and should be considered when interpreting results based on divergent reference genome quality. However, it is unlikely that this explains the surprising difference between mouse and rat cells, since the reference genome for these species is about as good as that of the human.

## 5. Conclusion

Here we show that mutagen-induced mutational burden in primary somatic cells, a direct measure of DNA repair error rate, is negatively correlated with maximum lifespan across 10 species. While our results only show a modest inverse correlation, the findings nevertheless add to previous evidence that genome instability is a primary hallmark of aging^[1]^, with DNA damage repair^[7,23]^ and somatic mutation rate^[14]^ being major determinants of species-specific life span. Utilizing our error-corrected single molecule sequencing method, we demonstrate that we can assess *de novo* SNVs across species without prior single cell isolation or clonal expansion. This opens new possibilities for further studies involving more species and different mutagens to test multiple DNA repair pathways. Of note, this same procedure can be used in testing human specimens and patient-derived samples as only a few nanograms of gDNA are required. With costs of sequencing further dropping, this will allow for pre- and post-treatment analysis of human samples to elucidate the impact of drugs or lifestyle interventions on individual health span.

## Supplementary Material

supplementary material

The supplementary material for this article is available at: Supplementary materials.

## Data Availability

All raw sequencing data has been deposited into NCBI SRA under accession number PRJNA1452926.
