# Supplementary material for: Mutagen-induced somatic mutation rate in primary mammalian cells in relation to maximum life span
Source: Geromedicine. Author manuscript; Available in PMC 2026 Jun 4. (PMC13233046; doi:10.70401/geromedicine.2026.0023)
Supplement: supplementary material [file NIHMS2180532-supplement-supplementary_material.pdf]

https://doi.org/10.70401/Geromedicine.2026.0023

Supplementary information

Mutagen-induced somatic mutation rate in primary mammalian cells in relation to maximum life span

Johanna Heid, Shixiang Sun, Julia Ablaeva, Moonsook Lee, Zhengdong Zhang, Vera Gorbunova, Andrei Seluanov, Alexander Y. Maslov, Jan Vijg

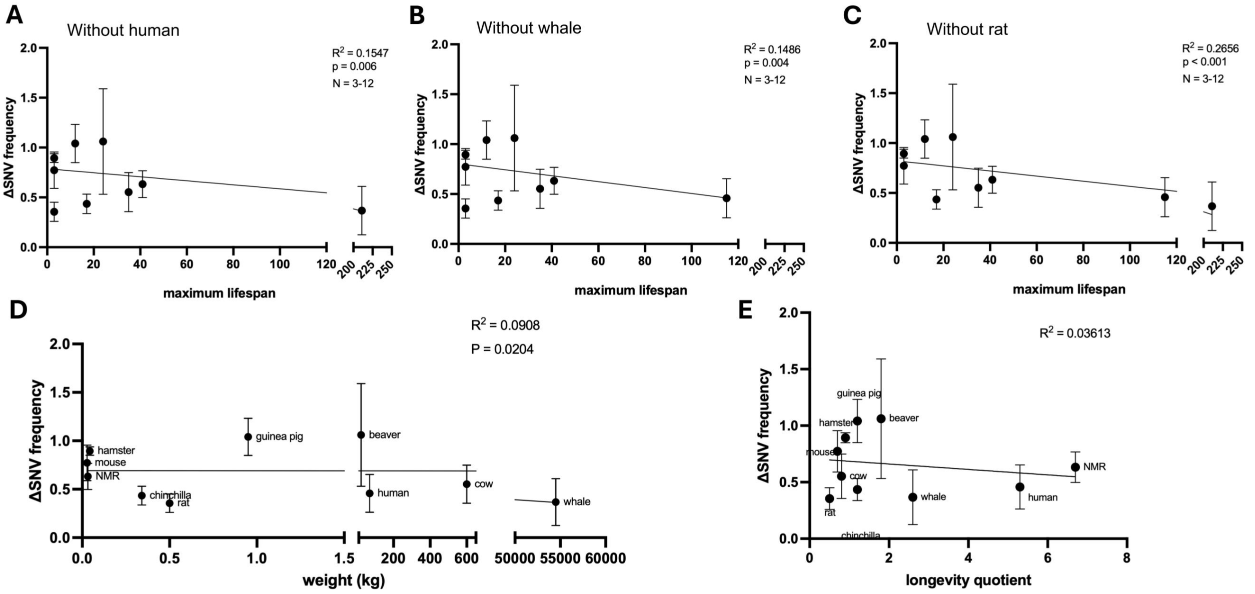

Figure S1. Correlation of ΔSNV with (A-C) lifespan, (D) body weight or (E) longevity quotient. SNV: single nucleotide variant.

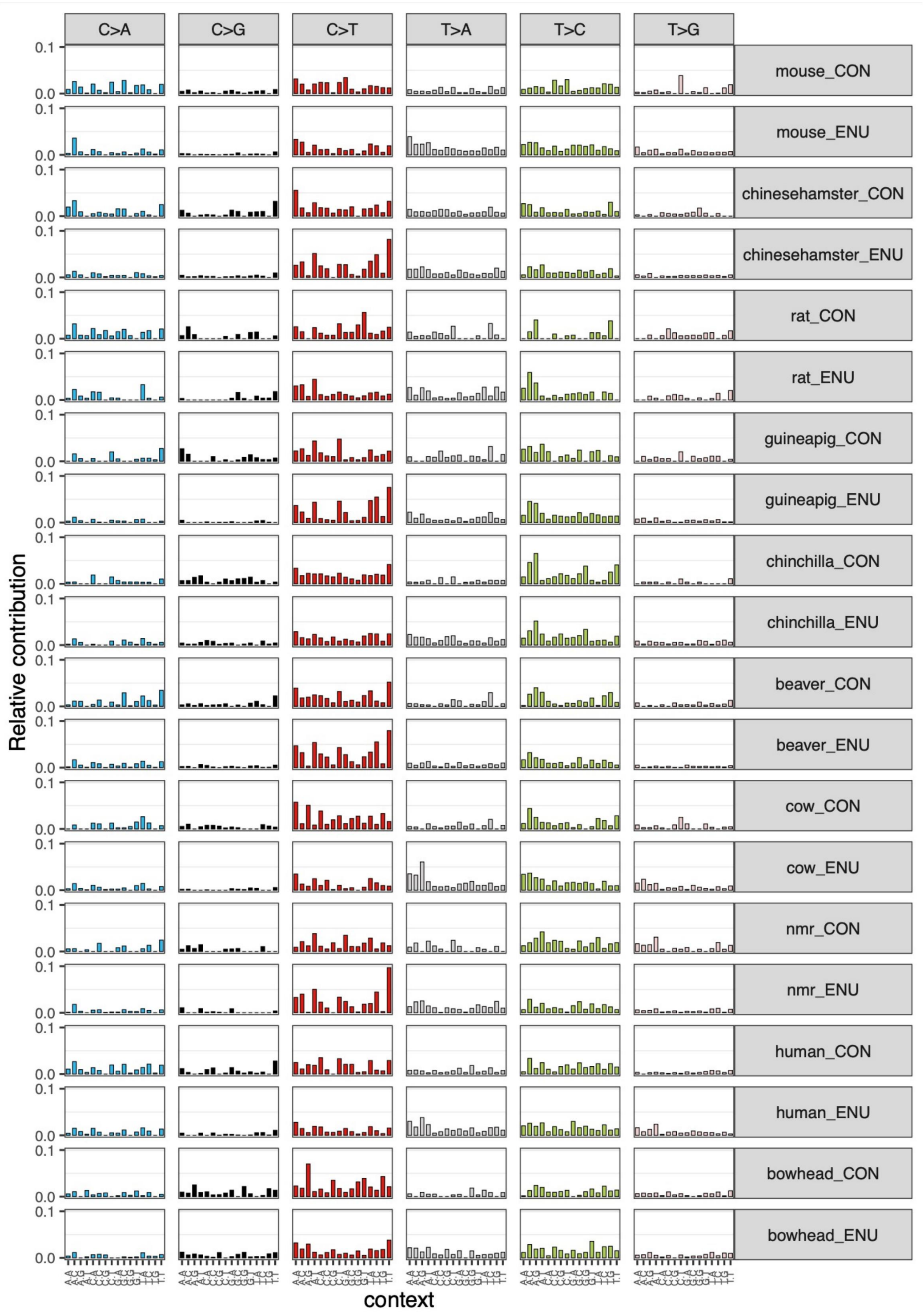

Figure S2. Relative contributions of somatic mutations in CON and treated (ENU) groups (A). The x-axis displays the mutational profiles following the standard 96 mutation type classification used in COSMIC, which incorporates the six base substitution categories and the immediate 5' and 3' nucleotide context, ordered by A, C, G, and T. CON: control; ENU: N-ethyl-N-nitrosoure; COSMIC: catalogue of somatic mutations in cancer.

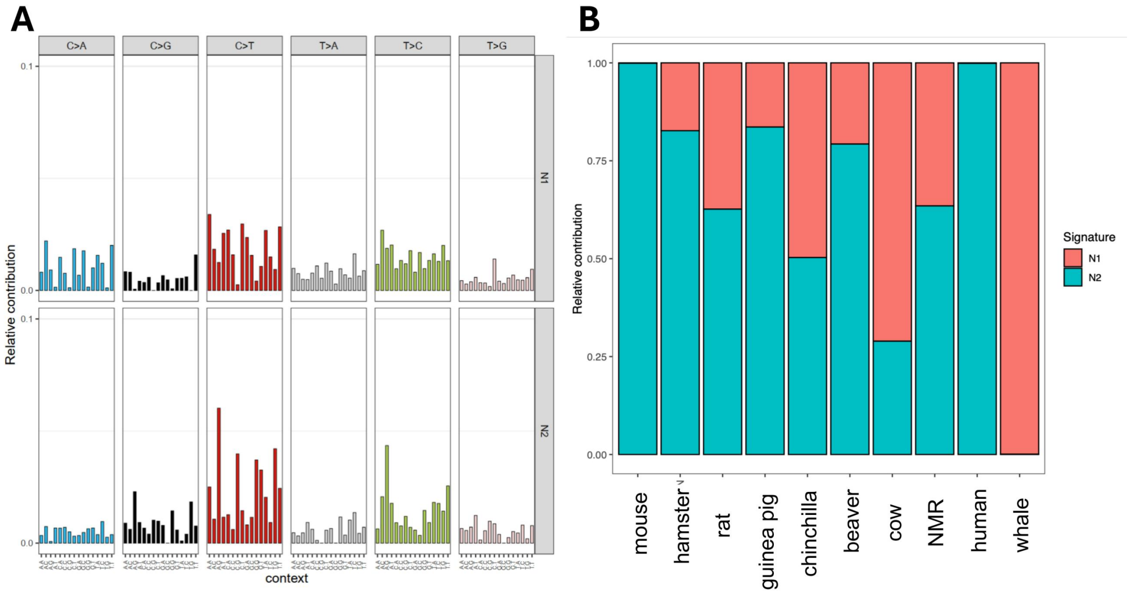

Figure S3. (A) Signature comparison of controls across species: Two *de novo* signatures are identified; (B) The contribution of two signatures for all surveyed species.

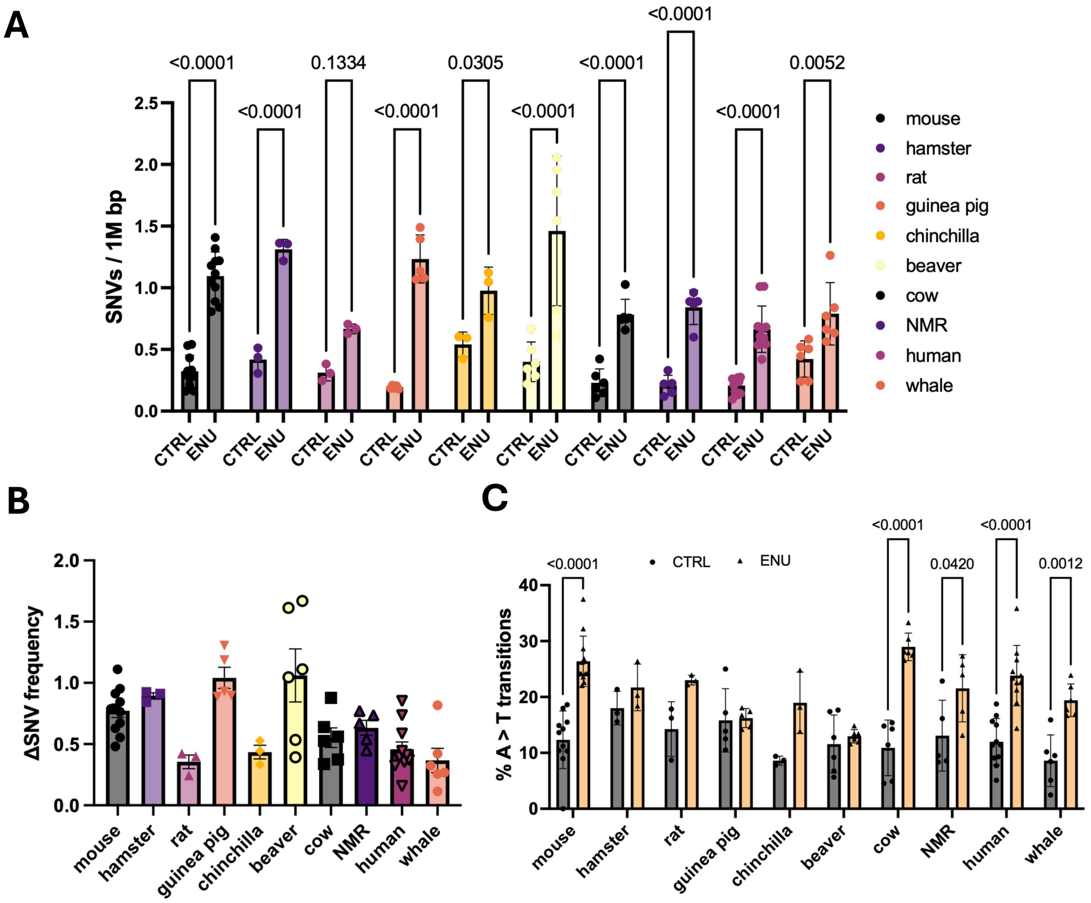

Figure S4. ENU treatment enriches A > T transitions across species. (A) The mutational spectra shift with ENU treatment; (B) In all species, a higher percentage of A > T transitions can be observed in cells treated with ENU compared to control cells; (C) The difference in mutational burden between control and ENU-treated cells is expressed by ΔSNV. ENU: N-ethyl-N-nitrosoure; SNV: single nucleotide variant.

Table S1. Maximum lifespan, weight and longevity quotient used for calculations.

Species	Max. lifespan (yrs)	Weight (kg)	LQ
Mouse	3	0.027	0.7
Hamster	3	0.043	0.9
Rat	3	0.5	0.5
Guinea pig	12	0.95	1.2
Chinchilla	17	0.341	1.2
Beaver	24	23	1.8
Cow	35	600	0.8
Naked mole rat	41	0.032	6.7
Human	115	70	5.3
Bowhead whale	211	54,500	2.6
